# Evaluation of uptake of the cytostatic methotrexate in *Elliptio complanata* mussels by LC–MS/MS

**DOI:** 10.1007/s11356-022-19064-7

**Published:** 2022-02-10

**Authors:** Sylvie Poirier Larabie, Martin Jutras, Grégoire Leclair, Isabelle St-Jean, Christine Kleinert, François Gagné, Christian Gagnon

**Affiliations:** 1 Environment and Climate Change Canada, Aquatic Contaminants Research Division, 105 McGill St., 8th floor, Montréal, Québec H2Y 2E7 Canada; 2 Faculté de Pharmacie, Université de Montréal, Plateforme de biopharmacieC.P. 6128, succ. Centre-ville, Montréal, Québec H3C 3J7 Canada

**Keywords:** Methotrexate, *Elliptio complanata* mussel, Liquid chromatography/tandem mass spectrometry, Uptake, Cytostatic drug

## Abstract

Aquatic organisms are continuously exposed to emerging contaminants coming from urban effluents of wastewater treatment plants. The contamination of surface water by those effluents poses a number of environmental risks, and pharmaceuticals are part of this class of effluent contaminants. Various classes of pharmaceuticals are not treated by wastewater treatment plants and anticancer drugs are part of them. The chemotherapy drug methotrexate (MTX) is an emerging contaminant and its growing use with the increase in cancer cases worldwide raises potential risk to aquatic organisms exposed to effluent discharges. However, chemical analyses in exposed freshwater aquatic organisms for ecotoxicological studies are rarely available and no studies have been done yet to accompany ecotoxicological data of exposed filter-feeding organisms. The purpose of this study was to develop a specific and sensitive analytical LC–MS/MS method for the quantification of methotrexate uptake in mussels exposed at different concentrations of the drug. A solid/liquid extraction followed by solid phase extraction (SPE) using an MCX phase purification scheme was optimized. The optimal recovery of 65% and matrix effect of 38% allowed to achieve a limit of quantification of 0.25 ng g^−1^, with an accuracy of 99–106%, a precision of no more than 3% RSD, and linearity ranging from 0.25 to 25 ng g^−1^. This methodology was tested with mussels exposed for 96 h at different concentrations (4 to 100 µg L^−1^) of MTX. The data revealed tissue uptake at concentrations ranging from 0 to 2.53 ng g^−1^. This suggests that this drug has low uptake potential and this methodology could be used to examine tissue levels of this drug in organisms continuously exposed to urban pollution.

## Introduction

Surface waters are a receptacle for runoff and urban effluents that release existing and emerging contaminants. Many pharmaceuticals have been found in surface waters (Bean et al. 2018; Brausch and Rand. 2011; Christen et al. 2010; Corcoran et al. 2010; Fatta-Kassinos et al. 2011; Richardson and Bowron 1985) and are still considered as emerging contaminants, since new ones are commercialized regularly and many studies need to be done to determine their presence and fate once released in the environment. Since the wastewater treatment plants are not configured to eliminate these contaminants, they release these compounds on a continuous basis and seemingly independent of the treatment type, whether it is a physicochemical primary treatment, biological treatment with activated sludge under aerobic conditions, or facultative and aerated lagoons, (Gagnon and Lajeunesse. 2012; Guerra et al. 2014) although advanced oxidation systems were previously shown to degrade them more effectively. Several classes of pharmaceuticals have been detected (from few ng L^−1^ to several µg L^−1^) in surface waters such as anti-inflammatory, antibiotics, antidepressants, β-blockers, anti-lipidemic agents, contrasting agents, hormones, anti-epileptics (Berryman. 2011; Corcoran et al. 2010; Fatta-Kassinos et al. 2011; Liu et al. 2010; Segura et al. 2011, 2009), and more recently cytostatics (up to 17 ng L^−1^) (Azuma et al. 2015; Ferrando-Climent et al. 2014; Kosjek and Heath. 2011; Mahnik et al. 2004). These pharmaceuticals are not an exception in terms of fate in treatment plants, i.e., they are weakly retained or degraded at WWTPs and thus largely released in effluent receiving waters (Azuma et al. 2015; Česen et al. 2015; Haddad et al. 2015; Kosjek et al. 2013; Lenz et al. 2007; Mukherjee et al. 2020; Zhang et al. 2013). There have been only a few studies in which several wastewater treatments have been tested for cytostatics (Česen et al. 2015; Chen et al. 2019; Ferrando-Climent et al. 2014; Lutterbeck et al. 2015; Ofiarska et al. 2016; Russo et al. 2018). The results of these experiments demonstrate high variability in treatment efficacy for cytostatics, and treatment combinations are more effective than a single treatment. These studies demonstrated the complexity of treating cytostatic as no treatment plants are designed to treat all pharmaceuticals.

Cytostatics are antineoplastic drugs for the treatment of cancers. They are classified into various categories, such as antimetabolites, alkylating agents, antitumor antibiotics, and plants. These drugs are one of the most toxic drugs used in therapeutics (NIOSH. 2016; Zounkova et al. 2010; Zounková et al. 2007). According to the WHO (WHO. 2021) on August 13th, 2021, cancer is one of the leading causes of death worldwide, causing 9.2 million deaths in 2018 and this number is estimated at 22 million by 2030. Therefore, the use of cytostatic to treat cancer by chemotherapy will likely continue to increase, unless other treatments are developed. Since WHO estimates cancer will continue to increase, it can be assumed that the amount of cytostatic will also increase in the effluent-receiving aquatic environment, likely affecting ecosystems. Hence there is need for robust methods to determine the exposure of these anthropogenic contaminants to aquatic organisms.

Little is known on their environmental fate in aquatic ecosystems (Česen et al. 2015; Heath et al. 2020; Lutterbeck et al. 2020; Mukherjee et al. 2020). The fate and distribution of cytostatic in environmental compartments can be, at some extent, predicted by their physicochemical properties such as solubility, dissociation constant (pKa), vapor pressure, n-octanol/water distribution coefficient (D_ow_) from which are derived octanol–water partition coefficient (K_ow_) and organic carbon partition coefficient (K_oc_), bioconcentration factor (BCF), and chemical structures (Heath et al. 2020; Kosjek and Heath. 2011). The sorption and the affinity of pharmaceuticals to organic matter and by extension their bioaccumulation are estimated mainly by the K_ow_ and the solid-water distribution coefficient (K_d_). Biodegradability, adsorption onto sludge and sediments, direct photolysis, and indirect photolysis of some cytostatic were reported (Heath et al. 2020; Kosjek and Heath. 2011). The chemical structure of vinblastine, vincristine, doxorubicin, epirubicin, daunorubicin, and mitoxantrone as well as plant alkaloids (actinomycin D, doxorubicin) and antitumor antibiotics (doxorubicin, bleomycin) favors their adsorption on organic matter as sewage sludge and sediments and thus has a bioaccumulation potential (Heath et al. 2020; Kosjek and Heath. 2011). Some other cytostatics such as cytarabine, gemcitabine, 5-fluorouracil, capecitabine, and methotrexate have been determined to be biodegradable (Heath et al. 2020; Kosjek and Heath. 2011).

To date, the studies indicate that since most cytostatic drugs are polar and persistent, this conducts to their high solubility and mobility in surface water. Partial degradation of those active substances must be considered in environmental assessments as well. For example, methotrexate biodegrades in its active metabolite 7-hydroxymethotrexate which has the same cytostatic mechanism as methotrexate (Białk-Bielińska et al. 2017; Kiffmeyer et al. 1998). The metabolite does not appear to biodegrade; in turn, it is thus considered as persistent in water. To better understand the fate and effects of these emerging contaminants in the aquatic environment, several complementary fields of expertise can be used, such as analytical chemistry and ecotoxicology. Long-term ecotoxicity and genotoxicity studies using various species were examined for many cytostatics and various effects were determined with high sensitivity (Białk-Bielińska et al. 2017; Grzesiuk et al. 2019; Jureczko and Przystaś. 2019; Kovács et al. 2015; Parrella et al. 2015, 2014; Zounkova et al. 2010). For more comprehensive ecological assessments, several of these studies raised the necessity of measuring the concentration of cytostatic in the biota used for toxicity tests, providing insight of their uptake. Methods were published for the analysis of other pharmaceuticals than cytostatics in mussel. For example, Bayen et al. (2015) have developed a screening method for the analysis of 44 pharmaceuticals with relative recoveries ranging from 26 to 163% and matrix effects ranging from 4 to 417%, resulting in detecting only trace levels of six compounds in sea water mussels. Another team (Núñez et al. 2016, 2015) developed a QuEChERS extraction, LC–MS/MS analytical method for the analysis of seven pharmaceuticals in bivalves resulting in apparent recoveries ranging from 35 to 77% with limit of quantification from 5 to 100 ng g^−1^. Alvarez-Muñoz et al. (2021) also developed a QuEChERS extraction, LC–MS/MS analytical method resulting in limit of quantification from 0.78 to 68 ng g^−1^ and apparent recovery from 16 to 134%. Mijangos et al.’s (2019) team developed a 41 multiclass organic pollutants with multistep extraction method with focused ultrasound liquid extraction (FUSLE) followed by solid phase extraction (SPE), analyzed by LC–MS/MS, resulting in apparent recovery values ranging from 71 to 126% and method detection limits ranging from 4 to 48 ng g^−1^. This method results in measuring only seven contaminants in sea water mussels. This demonstrates that multiclass methods offer variable results depending on the compound and cannot be applied in an ecotoxicology study where precision and accuracy of results are sought. Some methods were developed for the detection of cytostatic in other types of biological matrices like plasma and urine (Guo et al. 2007; Izzo et al. 2018). Several methods are published for the analysis of methotrexate in water (Kosjek et al. 2013; Rabii et al. 2014; Vaudreuil et al. 2020), in biological matrices such as mouse plasma and brain microdialysis samples (Guo et al. 2007) and human urine and plasma (Izzo et al. 2018). All those methods were carried out using LC–MS/MS and one of the major drawback of LC–MS analysis is the ionization competition by interferences from the co-extracted analytes such as proteins, phospholipids, and salts of the matrix (Boulard et al. 2020) causing either signal suppression or enhancement, especially in complex matrix such as biota. Unfortunately, those methods cannot be transposed directly for mussel tissue analysis since lipid and protein contents make the matrix effect quite different. The use of isotopically labeled internal standard is well recognized for counteracting the matrix effect, but it does not counteract for loss of sensitivity due to ion suppression. The best limit of quantification is thus obtained by the development of specific extraction and purification methods in the same mussel as those exposed since the proportion of proteins and phospholipids in the matrix influences the development of the method. This demonstrates the importance of developing robust and specific extraction and purification methods for ecotoxicological study where mussels are exposed to one cytostatics at a time, which requires to minimize matrix effect and optimize lower limit of quantification (LLOQ).

Exposure of *Elliptio complanata* mussels, an aquatic filter organism, to not only water component but also to everything that is adsorbed on suspended particles in water, was used in this study to better characterize ecosystem exposure to cytostatics, following a quantification in mussel tissues. Methotrexate is in the top 15 of the most used cytostatic drugs in the province of Quebec (Canada) and was selected for this study. This cytostatic is very polar with log K_ow_ of − 0.24 and thus has the potential to be retrieved in effluent-receiving waters. As a result, methotrexate has been found in a screening method at concentration up to 53 ng L^−1^ in surface water of St. Lawrence River (Canada) near effluent discharges (Rabii et al. 2014).

The purpose of this study was to develop a sensitive and specific analytical method to quantify methotrexate in mussel tissues. Mussels were chosen as the target organism given they are species at risk to urban pollutants because of their filter-feeding behavior and sessile lifestyle. Since previously published analytical methods are not applicable for the methotrexate analysis in mussel tissues, a new methodology was developed to determine methotrexate uptake in mussels.

## Methodology

### Materiel for mussel analysis

Standard of methotrexate (MTX) was purchased from Toronto Research Chemical (TRC). Isotopically labeled standard of methotrexate-d3 was purchased from Cerilliant and used as internal standards. Solid phase extraction (SPE) cartridges Oasis MCX 3 cc Vac Cartridge, 60 mg (cat no. 186000253), LC column Cortecs T3, 120 Å, 50 × 2.1 mm, 2.7 µm (cat no. 186008482), and Cortecs T3 VanGuard cartridge (cat no. 186008506) were obtained from Waters Corp. (Milford, MA, USA). Thermo Scientific N249944 96-well polypropylene V-bottom injection plate (cat no. 12–565-436), Thermo Scientific Nunc N276011 96-well pre-slit silicone cap mats (cat no. 12–565-570), falcon round-bottom polypropylene tubes, 14 mL (Corning C352059), borosilicate culture tubes 10 × 75 mm (cat no. 14–961-25), 15 mL polypropylene conical tube (cat no. 352196), and polyethylene transfer pipets (cat no. 13–711-7 M) were obtained from Fisher Scientific (Toronto, Ont., Canada). Optima grade acetonitrile (ACN) and methanol (MeOH), ACS grade formic acid (98%), phosphoric acid (85%) and ammonium hydroxide, ACS reagent 28–30% solution in water were obtained from Fisher Scientific (Toronto, Ont., Canada). Water was purified in-house by a Milli-Q ultrapure water system from Millipore (Bedford, MA, USA). Instruments used for this study were as follows: Sorvall Centrifuge, LC–MS/MS AB/SCIEX 4000 QTRAP (Agilent 1100 series HPLC system), UPLC-ESI-QTof Xevo G2-XS (Waters®).

### Preparation of standard solutions for mussel exposition and analysis

Standard solution of MTX for mussel exposition was prepared in DMSO at 1 mg mL^−1^. This standard solution was diluted with dechlorinated tap water at 3 different concentrations, 4 µg L^−1^, 20 µg L^−1^, and 100 µg L^−1^, in 10 L water basin for mussel exposure. The reference bucket contains a blank solution of DMSO in water proportional to standard solution prepared.

Individual stock solutions (1 mg mL^−1^) of each standard and internal standard (IS) were all prepared by dissolving appropriate amount of the standard in ACN/NH_4_OH (99:1, v/v) and stored at − 20 °C. From the primary stock solution at 1.0 mg/ml, intermediate stocks were prepared at 5000, 500, and 50 ng/mL in MeOH. A 7-point (non-zero) calibration curve was prepared by spiking appropriate volumes of intermediate stocks in blank matrix (1-g homogenized mussel wet weight) to generate standards at 25, 10, 5, 2.5, 1, 0.5, and 0.25 ng g^−1^. Three levels of QC samples (*n* = 2) were also prepared in blank matrix at 22.5, 7.5, and 0.75 ng g^−1^. Working internal standard of MTX-D_3_ was prepared at 1000 ng mL^−1^ in MeOH for the extraction of mussel tissue.

### Mussel exposition

Wild freshwater *Elliptio complanata* mussels were collected on June 2018 in pristine lakes in the Laurentians (Quebec, Canada). Prior exposure, the mussels were maintained for 6 months in 60-L aerated aquariums containing UV-treated and charcoal-filtered tap water at 15 °C, with 16-h-light/8-h dark cycle and fed with phytoplankton.

Groups of ten mussels were exposed to 10 L of standard solution diluted in dechlorinated water at 3 different concentrations, 4 µg L^−1^, 20 µg L^−1^, and 100 µg L^−1^, in 20-L buckets lined with polyethylene bags under constant aeration. The exposition took place for 96 h at 15 °C under 16-h-light/8-h dark cycle, with medium renewal each day. The pH was constant at approximately 8. A reference bucket was also used containing dechlorinated water only. To allow mussel time to be exposed to MTX and initiate defense mechanisms, a 96-h exposure period was used. At the end of the exposure period, mussels were placed in 10-L aquarium filled with dechlorinated water for 2–6 h to allow certain depuration such as gut clean-up/egestion and tissue surface desorption.

### Method development for mussel analysis

#### Extraction and purification

**Fig. 1:**
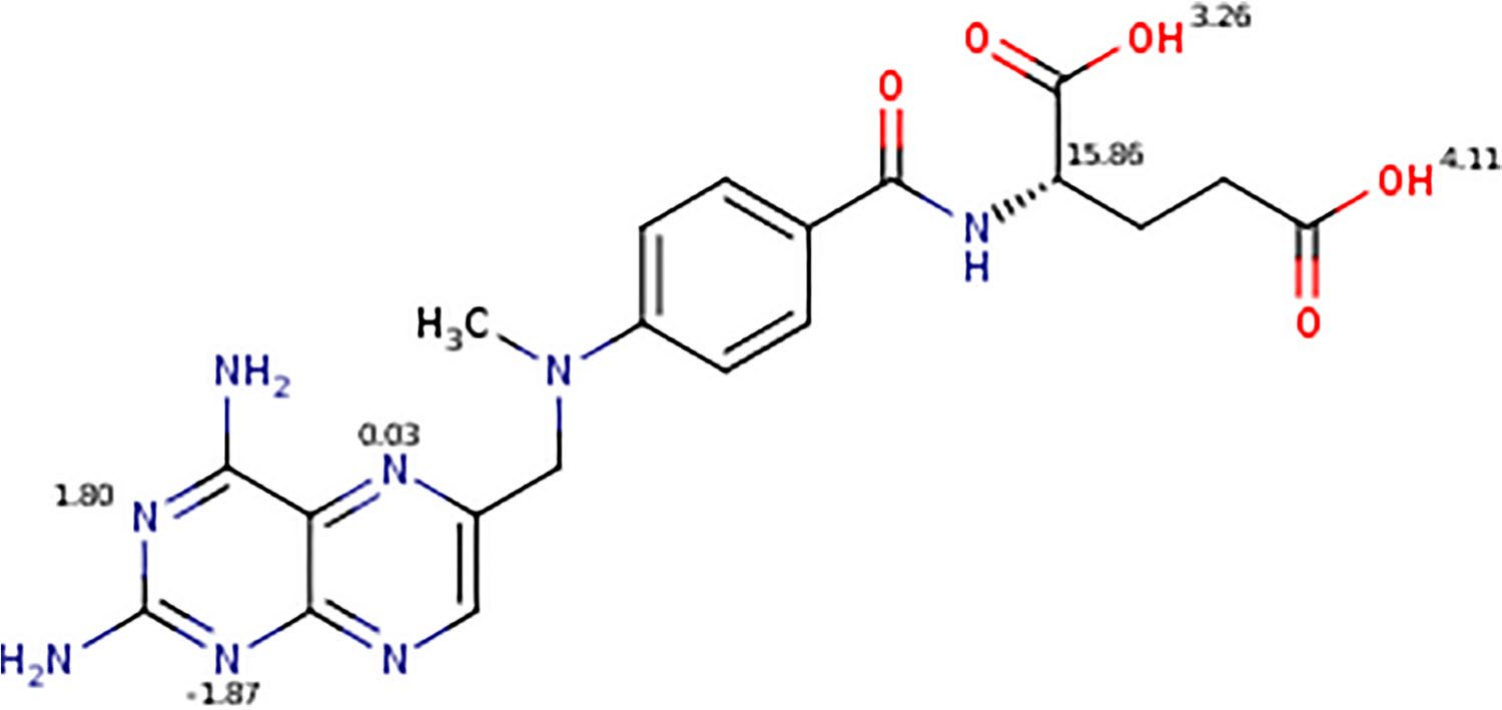
Structure of methotrexate with pKa

The method development for the extraction of MTX in mussel tissues was done in two steps, namely, a liquid–solid extraction with homogenisation of the tissue in an appropriate solvent, followed by purification with a solid phase extraction (SPE) on the solvent extract. MTX has two carboxyl moieties with pKa of 3.25 and 4.00 (Fig. 1), which are fully ionized (> 99%) above pH 6. The strongest basic pKa is 2.80, and MTX has one positive charge partially ionized at 85% at pH 2. Therefore, MTX could be extracted by SPE by using either strong anion (OASIS MAX) or cation (OASIS MCX) exchangers. Initial recovery experiments of MTX in homogenized *Elliptio complanata* mussels demonstrated better recovery with MCX when compared to MAX; MCX 60 mg/3 cc was thus used hereafter.

The type and pH of solvent for matrix homogenization of 1 g of mussel tissue spiked with MTX standard were investigated by comparing homogenization in 1:1 and 4:1 methanol:water mixtures, acidified with 0.2% phosphoric acid, or basified with 0.1% ammonium hydroxide (28–30%). Homogenization in 3 mL acidic solvent resulted in a large swollen matrix pellet after centrifugation, with less than 1 mL of supernatant available. Therefore, neutral or basified homogenization was pursued further. Basified homogenate did not result in better extraction recovery of MTX compared to neutral solvent, and 50% MeOH showed the same extraction recovery as 80% MeOH, but the supernatant was still turbid. Finally, using 4:1 acetonitrile:water mixture resulted in a clean supernatant which did not require any filtration step and raised the recovery percentage of MTX. The ACN was further basified with ammonium hydroxide, in case of there would be interactions from the two carboxyl groups of MTX with amino groups from the matrix. The supernatant (3 mL) was diluted with 9 mL of acidic water (0.1% phosphoric acid), mixed and subsequently loaded onto MCX 60 mg/3 cc SPE cartridges.

#### LC–MS/MS analysis

Optimization of chromatography was performed on two superficially porous C18 column. The 50 × 2 mm Cortecs T3 demonstrated better peak shape and height when compared to Kinetex 30 × 2 mm. Sensitivity of MTX was poor in negative ion mode. In ESI positive ion mode, MTX displayed twice the sensitivity (peak height) with MeOH acidified with formic acid when compared to ACN acidified with formic acid. However, during matrix effect tests, SPE extracts of *Elliptio complanata* mussels displayed stronger ion suppression with MeOH (approximately 50%) in the mobile phase when compared to ACN (approximately 25%) on a 5 cm Cortecs T3. Therefore a mixture of both solvent was chosen as the final organic solvent in mobile phase B, that is MeOH:ACN (75:25) containing 0.2% formic acid, for keeping sensitivity with the MeOH and to decrease the ion suppression with ACN. Calibration curve was plotted using peak area ratios analyte/deuterated internal standard versus nominal analyte concentration, using linear regression employing 1/*x* weighting.

As a complement of investigation, qualitative analyses were also performed for identifying potential metabolites in tissues (despite short exposure time to mussels). The metabolites of MTX in mussel extracts were screened for using a high-resolution mass spectrometry (HRMS) using QTof in the full scan MSe mode. The analysis was performed on a Waters ACQUITY UPLC system (autosampler I-Class with FTN, vacuum degasser, binary pumps, column heater) coupled with a Xevo G2-XS QTof mass spectrometer equipped with electrospray ionization (ESI) source in the positive ion mode. Acquisition parameters consist of a scan range from 100 to 1200 m/z with a scan time of 0.1 s. Collision energy was set to ramp from 6.00 to 25 eV. The chromatographic separation was performed on a Lunar Polar Omega C18 (2.1 × 100 mm, 1.6 µm, Phenomenex) column held at 50 °C. The mobile phase consisted of 0.1% formic acid in water (A) and 0.05% acetic acid in 95/5 methanol:water (B) delivered at a flow rate of 0.4 ml/min with a linear gradient program from 5% B to 75% B in 8 min.

The QTof was mass calibrated the day prior to the analysis of samples with 0.5 mM sodium formate in isopropanol-water (90:10 v/v). To ensure mass accuracy during MS analysis, the mass was corrected using leucine-encephalin at 200 ng/mL via a LockSpray interface at a flow rate of 10 µL/min, monitoring a reference ion ([M + H]^+^  = 556.2271) at intervals of 20 s. All data collected was acquired and processed using UNIFI (version 1.9.4) software. For the targeted screening of potential metabolites, a list of metabolic reactions was used (oxidation, demethylation, cleavage, desaturation, see Table 1) with a combination of these phase I biotransformations, using a mass extraction window (MEW) of 10 ppm (mass tolerance + / − 5 ppm).

**Table 1:** Summary of LC–ESI–MS/MS conditions

HPLC	Agilent 1100 Series				
MS/MS	AB/Sciex 4000 QTRAP				
Software	Analyst 1,6,2				
Ionisation mode	Turbo Electrospray in positive ion mode				
Scan mode	Multiple reaction monitoring (MRM) using 70 ms dwell time				
Analytes parameters	Compound	MRM	Declustering (V)	CE (V)	CXP (V)
	MTX	455 > 308	85	28	7.0
	MTX-d3	458 > 311	85	28	7.0
Source parameters	Gas temp (°C)		600		
	Gas flow 1 and 2		50 and 60		
	Curtain gas		25		
	Capillary (V)		5500		
Column temperature	45 °C				
Sample temperature	8 °C				
Column	Waters Cortecs T3, 120 Å, 50 × 2,1 mm, 2,7 µm				
Flow rate	0,7 mL/min				
Mobile phase	A: H2O + 0.2% formic acid				
	B: Methanol/Acetonitrile (75/25) + 0,2% formic acid				
Gradient	10 to 98% in 2 min, plateau at 98% for 0.6 min; equilibrate for 1.6 min at 10% B (total run time 4.2 min)				
Injection volume	6 µL				
Divert valve	Switch to MS between 1.3 and 2.4 min				

No metabolites related to MTX were detected in the sample extracts in the accurate mass XIC chromatograms of potential metabolites resulting from oxidation, demethylation, desaturation, cleavage, and a combination thereof.

#### Method validation and quality assurance

Using the final extraction and chromatographic conditions, the resulting process efficiency for MTX (combining extraction recovery of approximately 66% and ion suppression of approximately 44%) was in the range of approximately 37%. These values were determined more accurately during validation. In these conditions, with the use of the deuterated internal standard, it was possible to obtain a LLOQ of 0.25 ng MTX/g mussels. For validation experiments, drug-free *Elliptio complanata* mussels were used as blank matrix. The validation parameters included linearity, lower limit of quantification, accuracy and precision, extraction recovery, matrix effect, and selectivity. A 7-point (non-zero) calibration curve was prepared by spiking appropriate volumes of intermediate stocks in blank matrix (1-g homogenized mussel) to generate standards at 25, 10, 5, 2.5, 1, 0.5, and 0.25 ng/g. Three levels of QC samples (*n* = 4) were also prepared in blank matrix at 22.5, 7.5, and 0.75 ng/g. The calibration curve standard and QC plasma samples were treated as mentioned below. Also, three matrix blanks were extracted for selectivity, and three others for post-spike investigation of ion suppression.

## Results

### Development and validation of mussel extraction method

Final extraction was done on 1-g mussel aliquots (wet weight) to which were added 10 µL of working internal standard (MTX-d3 at 1000 ng/mL in MeOH) and 3 ml of acetonitrile:water (80:20) containing 0.05% NH_4_OH. Samples were homogenized for 10 s with Polytron. Tubes were centrifuged at 3200 rpm for 10 min. The sample extract was transferred by pouring into 15-mL polypropylene tubes, and 9 ml of 0.1% phosphoric acid in water was added. Samples were loaded using a vacuum of 2 psi on OASIS MCX SPE 60 mg/3 cc cartridges, which were previously conditioned with 2 ml MeOH, followed by 2 ml water containing 2% formic acid. This was followed by washing with 2 ml water containing 2% formic acid, and then with 1 ml MeOH. Vacuum was increased to 20 mm Hg and cartridges were dried for 1 min. Elution was performed with 2.5 mL of 6% ammonium hydroxide in MeOH into borosilicate glass tubes. Eluent was evaporated to dryness at 45 °C under nitrogen. Residue was redissolved in 150 µl methanol:water (20:80) containing 20 mM ammonium acetate. Tubes were vortex mixed for 5 s. Extracted samples were transferred into a shallow polypropylene injection plate and centrifuged for 10 min at 3200 rpm. The supernatant was transferred in a new polypropylene injection plate and covered with a silicone mat.

Chromatography was performed with a gradient elution mode of two mobile phases composed of H_2_O/0.2% formic acid and MeOH/ACN (75/25)/0.2% formic acid, MeOH raising the sensitivity and ACN lowering the matrix effect, onto a superficially porous Cortecs T3 column 50 × 2.1 mm, 2.7 µm (Waters) with mass spectrometry parameters set on the positive ion mode, with selective MRM scanning. A summary of LC–MS/MS conditions is found in Table 2.

**Table 2:** Transition m/z used for metabolites screening

Compound	MRM
MTX	455 > 308
MTX-d3	458 > 311
MTX desmethyl	441 > 294
MTX oxidative	471 > 324
MTX desmethyl/oxidative	457 > 310
DAMPA	326 > 175
DAMPA desmethyl	312 > 175
DAMPA oxidative	342 > 175
DAMPA oxidative	342 > 191
DAMPA desmethyl/oxidative	328 > 175
DAMPA desmethyl/oxidative	328 > 161

The linearity was observed on the range of the calibration curve 0.25 to 25 ng g^−1^ with an accuracy of all standard between 93 and 106% of the nominal value, using a linear fit employing a 1/*x* weighting (*R*^2^ = 0.9992); LLOQ results in accuracy of 104%; accuracy of all standards was between 93 and 106% of the nominal value. Accuracy and precision were evaluated with QC samples spiked at three concentration levels (0.75, 7.5, and 22.5 ng g^−1^) with four replicates at each level and calculated as the ratio of the regressed concentration over nominal concentration. The precision of QC concentration 0.75, 7.5, and 22.5 ng g^−1^ was respectively of 2.15%, 1.60%, and 0.77%. The accuracy of QC concentration 0.75, 7.5, and 22.5 ng g^−1^ was respectively of 106.5%, 105.5%, and 99.5%. The mean precision (% RSD) was better than 3% and the mean accuracy (% nominal) was within 99–107%. Extraction recovery (ER) was evaluated as the ratio of the peak area response of MTX in extracted spiked matrix blank to that of MTX in post-extraction spiked matrix blank at the same concentration. Extraction recovery was assessed at three QC levels (0.75, 7.5, and 22.5 ng g^−1^) using four replicates at each level. Matrix effect (ME) was evaluated as the ratio of the peak area response of MTX in post-extraction spiked matrix blank to that of MTX in neat solution in solvent at the same concentration. Average extraction recovery from mussel homogenate (*n* = 12, 3 concentrations) was 65 ± 13% for MTX and average matrix effect (ion suppression) due to extracted components was 38 ± 14%. The method was selective (*n* = 3 different *Elliptio* mussel sample) and sensitive enough to routinely measure MTX down to 0.25 ng g^−1^ as matrix interferences were lower than 10% of the LLOQ. The carryover was lower than 10% of the LLOQ at 0.25 ng g^−1^ (see Table 3 for a summary of validation results).

**Table 3:** Validation parameter results for MTX

Parameters	MTX
Linearity (concentration) (accuracy ± 15% nominal)	0.5–25 ng/g
Linearity (1/*x*; *R*^2^)	0.9992
LLOQ (Accuracy ± 20% nominal)	0.5 ng/g
Precision (3 QC low-mid-high; *n* = 4)	≤ 3%
Accuracy (3 QC low-mid-high; *n* = 4)	99–106%
Extraction recovery (3 QC low-mid-high; *n* = 4)	58–73%
Matrix effect (3 QC low-mid-high)	34–44%
Selectivity (% of LLOQ; *n* = 3)	≤ 10%
Carry over (% of LLOQ; solvent injected after HLOQ)	≤ 10%

### Results for mussels exposed to MTX

MTX concentrations in mussels were calculated with a calibration curve ranging from 0.25 to 25 ng g^−1^ using a linear fit employing a 1/*x* weighting (*R*^2^ = 0.9997). The accuracy of each point of the calibration curve used for the quantification of exposed mussel samples was between 94 and 106%. The QC samples used for quality control of the batch analysis at three concentrations resulted in accuracies between 70 and 104%. At QC low, 0.75 ng g^−1^, accuracies were 104% and 90.3%; at QC med, 7.5 ng g^−1^, accuracies were 89.7% and 79.6%; and at QC high, 22.5 ng g^−1^, accuracies were 70.0% and 81.1%. All concentrations measured in the mussel samples were below QC med.

**Fig. 2:**
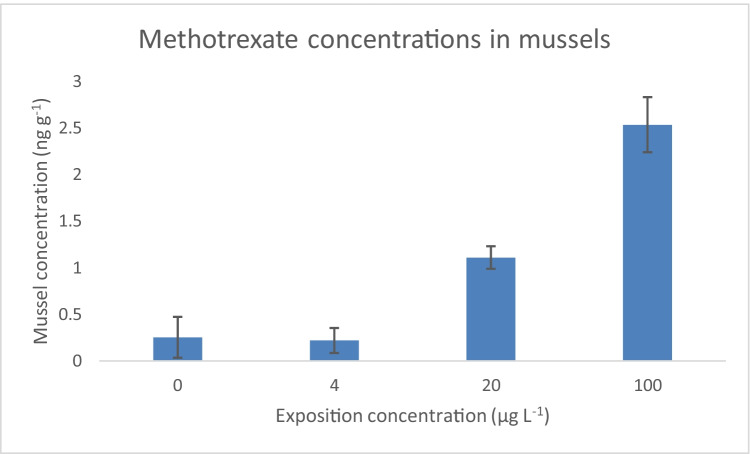
Concentrations of methotrexate in mussels for each concentration of exposure

Samples of *Elliptio complanata* mussels (*n* = 40) exposed at MTX by a group of ten mussels for each concentration were individually analyzed following the validated method (Table 3). In average, the group exposed to 4 µg L^−1^ of MTX had taken up concentrations of 0.219 ± 0.268 ng g^−1^ of MTX, while higher values of 1.11 ± 0.24 ng g^−1^ and 2.53 ± 0.59 ng g^−1^ were calculated for exposure to 20 and 100 µg L^−1^, respectively (Table 4). The reference group presented concentrations of 0.253 ± 0.439 ng g^−1^, since one blank out of them resulted with a concentration of 0.76 µg g^−1^, probably due to matrix effect or contamination, which means that the results of the group exposed to 4 µg L^−1^ become insignificant as the standard deviation is greater than the concentrations obtained in that group (Fig. 2).

**Table 4:** Results for MTX concentrations in mussels

Exposition to MTX	Concentration in mussels		Concentration factor
	Average	Standard deviation	
(µg/L)	(ng/g)	(ng/g)	(%)
0	0.253	0.439	ND
4	0.219	0.268	5.5%
20	1.109	0.242	5.5%
100	2.534	0.592	2.5%

## Discussion

The main objective of the MTX exposure experiment with mussels was to investigate short-term uptake and provide insights on the chronic exposure potential as a part of a broader investigation of ecotoxicological impacts. The toxicological effects of MTX were demonstrated by the increase in dehydrofolate reductase (DHFR) activity in the gonad and correlated with GST activity (Kleinert et al. 2021). Given that MTX is known inhibitor of DHFR activity, the observed induction represents an adaptive response of these mussels to maintain folate for purine (DNA) biosynthesis. Because pharmaceuticals are designed to circulate in the body and then be eliminated primarily through urine resulting in no concentration buildup in tissues, their bioaccumulation is not expected. This is seemingly the situation for mussels based on the present study. However, during chronic exposure such as that which occurs near municipal wastewater discharges, one can expect to find a constant concentration of pharmaceuticals in an organism. The proposed methodology is therefore of value to determine MTX uptake in mussels continuously exposed to municipal effluents. An analytical method has therefore been developed to quantify a common cytostatic, MTX, in exposed mussels since, to our knowledge, no method has been published on its quantification in organism tissues. The best limit of quantification was obtained by the development of specific extraction and purification methods in the same mussel tissues as the one exposed to MTX, dealing with matrix interferences. As the exposure concentrations ranged from 4 to 100 µg L^−1^, and concentrations in mussel were unknown, the method development was conducted to achieve the best LLOQ possible. Finally, a LLOQ of 0.25 ng g^−1^ was achieved. As expected, the measured concentrations in mussels were very low. In fact, half of the values obtained in mussels exposed at 4 µg L^−1^ were below LLOQ. All other results for mussel exposed at 20 and 100 µg L^−1^ were within the linearity range.

In comparison, some method developments and validations have been reported for the analysis of other pharmaceuticals in many kinds of mussel species (Álvarez-Ruiz et al. 2021; Bayen et al. 2015; Daniele et al. 2016; Mijangos et al. 2019; Núñez et al. 2016, 2015). The limit of quantification (LOQ) was typically in the range 5–125 ng g^−1^, with some exceptions lower than 1 ng g^−1^. Methods used for the determination of LLOQ are mostly based on S/N ≥ 10 or with Student’s *t*-test on mussel spiked at higher concentration than LLOQ; however, this method prevents checking the exactitude of the LLOQ at its real measured concentration. The determination of recovery is also mostly based on higher concentrations than environmentally relevant ones that might be found in mussels, and it is well-known that recovery can change drastically with concentration as it was demonstrated in our method development, when testing recovery with mussel spiked at 50 ng g^−1^ resulted in 70% and decreased below 50% at a spike concentration of 1.25 ng g^−1^. Matrix effect is probably the key in variation of recovery based on the concentration, the greater the matrix effect, the higher will be the LLOQ and the greater the impact on the total recovery. Also, the challenge of extracting drugs in tissue representing the reality of uptake drugs cannot be assumed to be mimicked at 100% by spiked QC samples (Xue et al. 2012). Mussel tissue quantity used for the extraction may also have an impact on matrix effect. Many methods in pharmaceutical research use 50 to 200 mg of tissue, for their extraction and it surely minimizes the matrix effect (Yang et al. 2005), but the concentration in the tissue must be high enough to be detected. Even in fish, methods for usual pharmaceuticals have been published with 50–100 mg of tissue (Boulard et al. 2020).

In addition, it is noted that the percentage of the exposed concentration found in mussels is inversely proportional to the exposure concentration (Fig. 2) that is about 5% for mussels exposed to both 4 µg L^−1^ and 20 µg L^−1^, but 2.5% for highest exposure concentration 100 µg L^−1^. This could indicate a saturation in mussel tissues with an increase in concentration of MTX and no bioaccumulation of the substance. From another point of view, mussels have the capacity to close their shells when they feel unsafe unlike other aquatic organisms such as fish and this could also affect tissue levels of MTX. Also, MTX is negatively ionized at both carboxylic acid groups at the exposure pH of 8, which makes it even more hydrophilic, which could explain, in part, the low concentrations found in mussel tissues. For comparison purpose, Rabii et al. (2014) have found MTX concentrations of 13 to 53 ng L^−1^ in effluent samples in Montreal area which are much lower than the exposed concentrations used in this study. Since the concentrations found in mussels exposed at 4 µg L^−1^ were not significant, it can be inferred that the MTX concentrations of mussels exposed in natural waters would be lower and likely not quantifiable. Published data on the uptake of cytostatics in tissues are limited, but Meredith-Williams et al. (2012) have published data on uptake of 5-fluorouracil in *Gammarus pulex*, with a radiolabeled 5-fluorouracil technique using exposure concentrations in the range of 0.2 to 0.8 µmol L^−1^ (26 to 104 µg L^−1^) and reported tissue concentrations with average concentration of about 1000 pmol g^−1^ (13 µg g^−1^) after 48 h exposure. However, such measurements using radiolabeled compound must be considered with caution as the technique does not differentiate between the parent drug and the metabolites (Xue et al. 2012), and since 5-fluorouracil has many of them, care must be taken when interpreting the results.

At the same time, the opportunity was taken to investigate if MTX was metabolized in the mussel, by doing some additional mass spectrometry experiments, using a UPLC-ESI-QTof Xevo G2-XS (Waters). No metabolites related to MTX have been detected in accurate mass XIC chromatograms of potential metabolites resulting from oxidation, demethylation, desaturation, cleavage, and a combination thereof. Analysis of metabolites was also performed in MRM mode on the LC–MS/MS, using MRM transitions corresponding to MTX oxidation, MTX demethylation, MTX oxidation/demethylation, DAMPA metabolite, DAMPA oxidized, DAMPA demethylated, and DAMPA oxidized/demethylated. Even in the selective and sensitive MRM mode, no peaks were detected at the transitions specified above.

## Conclusion

In conclusion, a new method for the determination of MTX in mussel tissue was developed and validated. The limit of quantification (0.25 ng g^−1^) achieved through good recovery (65%) and matrix effect (38%), as well as the precision (≤ 3% RSD) and accuracy (99–106%) never reached in mussel tissues, allowed the analysis of mussels exposed to MTX. This method was used to evaluate uptake in mussels exposed to MTX at concentrations ranging from 4 to 100 µg L^−1^. The results demonstrated that short-term exposure of mussel to MTX leads to low concentration in tissue, but chronic exposure due to constant wastewater releases is still a potential environmental risk that needs further investigations. To our knowledge, this is the first published method for the analysis of MTX in biota which addresses a gap in environmental sciences. Since new cancer treatments are continuously developed, there is a need to adapt this method to other cytostatic drugs as it will allow to document their dose response in aquatic organisms.

## Data Availability

N/A, data supporting the results reported in the article can be found in the figures and tables included in this paper.
